# Flat-topped papules coalescing into plaques in the axillae

**DOI:** 10.1016/j.jdcr.2025.04.028

**Published:** 2025-05-09

**Authors:** Christian F. Guerrero-Juarez, Catherine Emerson, Kyle T. Amber

**Affiliations:** a Carle Illinois College of Medicine, University of Illinois, Urbana-Champaign, Urbana, Illinois; b Department of Dermatology, Rush University Medical Center, Chicago, Illinois; c Piedmont Plastic Surgery and Dermatology, Hickory, North Carolina

**Keywords:** flexural skin, hyperpigmented plaque, papule, skin of color, syringoma, violaceous plaque

A 48-year-old woman with Fitzpatrick skin type IV presented for evaluation of lesions in the axilla that developed around the time of puberty. These were occasionally irritated with hair removal and certain antiperspirants. Physical examination revealed violaceous, thin, flat-topped papules coalescing into reticulated plaques in both axillae (Fig 1). She denied progressive hyperpigmentation or any lesions in the intergluteal and inframammary folds, neck, trunk, or inner arms or thighs. Punch biopsy showed scattered dilated glandular structures containing eosinophilic material, epithelial cords, and comma-shaped tubules lined by 2 layers of monomorphous small epithelial cells embedded in the reticular dermis (Fig 2).

**Fig 1:**
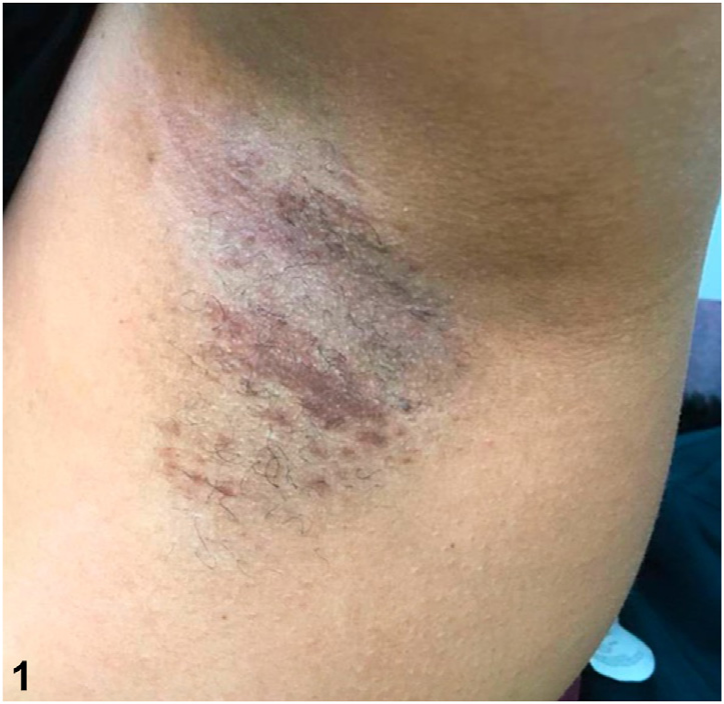

**Fig 2:**
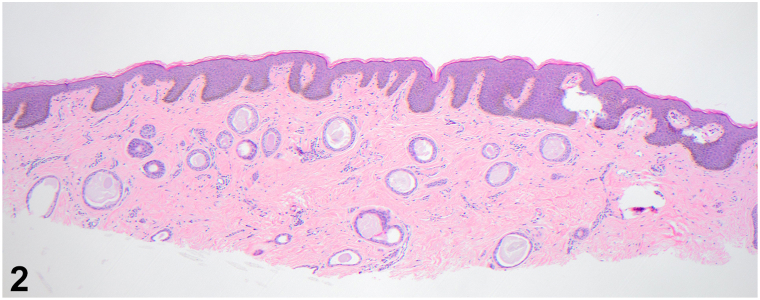

**Question 1: What is your diagnosis? Representative histological micrographs for a punch biopsy are shown in** Fig 2

A. Dowling-Degos disease
B. Galli-Galli disease
C. Fox-Fordyce disease
D. Syringoma
E. Acanthosis nigricans

**Answers:**

A. Dowling-Degos disease – Incorrect. It is characterized by reticular hyperpigmentation in flexural sites composed of lentigo-like brown macules and papules with variable hyperkeratosis. Histologically, it is characterized by increased pigmentation in the stratum basale and downward elongation of rete ridges. There is dermal infiltrate of melanophages and perivascular lymphohistiocytes.
B. Galli-Galli disease – Incorrect. It presents with erythematous macules and reticulated, hyperpigmentary changes with a pruritic papular eruption in flexural sites. Histologically, it presents with suprabasal acantholysis and dyskeratotic keratinocytes in the stratum spinosum and granulosum. In the inflammatory phase, there is papillary dermal lymphocytic, histiocytic, and eosinophilic infiltrate. In the noninflammatory phase, there is minimal acantholysis without dermal inflammation.
C. Fox-Fordyce disease – Incorrect. It presents with a pruritic, papular eruption in skin sites rich in apocrine glands, such as the axillae and anogenital regions. Histologically, there is hyperkeratosis with plugging of the infundibulum and excretory duct of the apocrine gland. There is lymphocytic and foamy histiocytic infiltration.
D. Syringoma – Correct. It presents as reticulated plaques composed of small confluent papules in intertriginous sites. Histologically, they are composed of layered epithelial cells that may form nests, cords, or tubules with a characteristic comma-like tail with mild fibroplasia of the surrounding stroma.^1^
E. Acanthosis nigricans – Incorrect. It is a velvety, darkening, and thickening of intertriginous sites with poorly defined borders. It is mostly associated with diabetes mellitus, insulin resistance, systemic glucocorticoid, and oral contraceptive use. It is rarely a sign of malignancy. Histologically, there is papillomatosis, hyperkeratosis, and hyperpigmentation, without dermal infiltrate.

**Question 2: Which of the following immunostainings would you expect to be negative in this condition?**

A. Cytokeratin AE1/AE2
B. SRY-box transcription factor 10
C. Carcinoembryonic antigen
D. Epithelial membrane antigen
E. Cytokeratin 5

**Answers:**

A. Cytokeratin AE1/AE2 – Incorrect. Though not specific, syringomas stain positive for AE1/AE2. These are monoclonal antibodies that detect several cytokeratins. AE1 detects cytokeratins 10, 14, 15, 16, and 19; whereas AE3 detects 1, 2, 3, 4, 5, 6, 7, and 8.^1^
B. SRY-box transcription factor 10 – Correct. SRY-box transcription factor 10 is a neural crest transcription factor and marks melanocytes and neural cells. It stains several sweat gland tumors but not syringomas.^2^
C. Carcinoembryonic antigen – Incorrect. Carcinoembryonic antigen stains the inner cells and inner secretion within the lumina. The duct outer cells and epithelial strands stain negatively.^3^
D. Epithelial membrane antigen – Incorrect. Epithelial membrane antigen stains the peripheral cells of dermal ducts and the intraepidermal duct. The duct luminal cells stain negatively.^3^
E. Cytokeratin 5 – Incorrect. Cytokeratin 5 stains the outer cells of the dermal duct and the lower intraepidermal ducts. The duct luminal cells stain negatively.^3^

**Question 3: Which of the following therapies is least likely to be effective in treatment?**

A. Retinoids
B. Corticosteroids
C. Atropine
D. Electrodessication
E. Laser

**Answers:**

A. Retinoids – Incorrect. Topical retinoids have been reported as a viable treatment option for syringomas—though with variable success. There is evidence for successful management of vulvar and acral syringomas; whereas intertriginous syringomas do not respond to retinoids.
B. Corticosteroids – Correct. Syringomas usually do not response to topical corticosteroids.
C. Atropine – Incorrect. Disruptive eruptive syringomas often respond to topical atropine, leading to disappearance of pruritus and a discrete reduction in the size of lesions. However, atropine is primarily used for symptomatic, and not asymptomatic, syringomas.^4^
D. Electrodessication – Incorrect. Electrodessication is a commonly used and reliable method for eliminating syringomas, with extensive reports demonstrating lack of long-term adverse effects and low recurrence rates.^5^
E. Laser – Incorrect. Ablative and nonablative fractional laser therapies are effective and safe treatment modalities for the management of syringomas.

## Conflicts of interest

None disclosed.
